# GASOL: Gas Analysis and Optimization for Ethereum Smart Contracts

**DOI:** 10.1007/978-3-030-45237-7_7

**Published:** 2014-12-15

**Authors:** Elvira Albert, Jesús Correas, Pablo Gordillo, Guillermo Román-Díez, Albert Rubio

**Affiliations:** 8 Johannes Kepler University, Linz, Austria; 9 University of Birmingham, Birmingham, UK; 10 Instituto de Tecnología del Conocimiento, Madrid, Spain; 11 Complutense University of Madrid, Madrid, Spain; 12 Universidad Politécnica de Madrid, Madrid, Spain

## Abstract

We present the main concepts, components, and usage of Gasol, a Gas AnalysiS and Optimization tooL for Ethereum smart contracts. Gasol offers a wide variety of *cost models* that allow inferring the gas consumption associated to selected types of EVM instructions and/or inferring the number of times that such types of bytecode instructions are executed. Among others, we have cost models to measure only storage opcodes, to measure a selected family of gas-consumption opcodes following the Ethereum’s classification, to estimate the cost of a selected program line, etc. After choosing the desired cost model and the function of interest, Gasol returns to the user an upper bound of the cost for this function. As the gas consumption is often dominated by the instructions that access the storage, Gasol uses the gas analysis to detect under-optimized storage patterns, and includes an (optional) automatic optimization of the selected function. Our tool can be used within an Eclipse plugin for Solidity which displays the gas and instructions bounds and, when applicable, the gas-optimized Solidity function.

## 1 Introduction and Main Applications

Ethereum [27] is a global, open-source platform for decentralized applications that has become the world's leading programmable blockchain. As other blockchains, Ethereum has a native cryptocurrency named *Ether*. Unlike other blockchains, Ethereum is programmable using a Turing complete language, i.e., developers can code smart contracts that control digital value, run exactly as programmed, and are immutable. A smart contract is basically a collection of code (its functions) and data (its state) that resides at a specific address on the Ethereum blockchain. Smart contracts on the Ethereum blockchain are metered using *gas*. Gas is a unit that measures the amount of computational effort that it will take to execute each operation. Every single operation in Ethereum, be it

*This work was funded partially by the Spanish MCIU, AEI and FEDER (EU) projects RTI2018-094403-B-C31 and RTI2018-094403-B-C33, the MINECO and FEDER (EU) projects TIN2015-69175-C4-2-R and TIN2015-69175-C4-3-R, by the CM projects P2018/TCS-4314 and S2018/TCS-4339 co-funded by EIE Funds of the EU and by the UCM CT27/16-CT28/16 grant.

†The software and dataset used during the current study are available at 10.6084/m9.figshare.11876697

a transaction or a smart contract instruction execution, requires some amount of gas. The gas consumption of the Ethereum Virtual Machine (EVM) instructions is spelled out in [27]; importantly, instructions that use replicated storage are gas-expensive. Miners get paid an amount in *Ether* which is equivalent to the total amount of gas it took them to execute a complete operation. The rationale for gas metering is threefold: (i) Paying for gas at the moment of proposing the transaction prevents the emitter from wasting miners computational power by requiring them to perform worthless intensive work. (ii) Gas fees disincentive users to consume too much of replicated *storage*, which is a valuable resource in a blockchain-based consensus system (this is why storage bytecodes are gas-expensive). (iii) It puts a cap on the number of computations that a transaction can execute, hence prevents DoS attacks based on non-terminating executions.

Solidity [13] is the most popular language to write Ethereum smart contracts that are then compiled into EVM bytecode. The Solidity compiler, **solc**, is able to generate only *constant* gas bounds. However, when the bounds are *parametric* expressions that depend on the function parameters, on the contract state, or on the blockchain state (according to the experiments in [8] this happens in almost 10% of the functions), named **solc**, returns $\infty$ as gas bound. This paper presents Gasol [6], a resource analysis and optimization tool that is able to infer parametric bounds and optimize the gas consumption of Ethereum smart contracts. Gasol takes as input a smart contract (either in EVM, disassembled EVM, or in Solidity source code), a selection of a cost model among those available in the system (c.f. Section 2), and a selected public function, and it automatically infers *cost upper bounds* for this function. Optionally, the user can enable the gas optimization option (c.f. Section 3) to optimize the function w.r.t. storage usage, a highly valuable resource. Gasol has a wide range of applications: (1) It can be used to estimate the gas fee for running transactions, as it soundly over-approximates the gas consumption of functions. (2) It can be used to certify that the contract is free of out-of-gas vulnerabilities, as our bounds ensure that if the gas limit paid by the user is higher than our inferred gas bounds, the contract will not run out-of-gas. (3) As an attacker, one might estimate, how much *Ether* (in gas), an adversary has to pour into a contract in order to execute an out-of-gas attack. Also, attacks were produced by introducing a very large number of underpriced bytecode instructions [23]. Our cost models could allow detecting these second type of attacks by measuring how many instructions will be executed (that should be very large) while its associated gas consumption remains very low. (4) As we will show in the paper, the gas analysis can be used to detect gas-expensive fragments of code and automatically optimize them.

## 2 Gas Analysis using Gasol

Figure 1 overviews the components of the Gasol tool [6]. The programmer can use Gasol during the software development process from its Eclipse plugin that allows selecting the cost model of interest and the function to be analyzed and/or optimized from the Outline. This selection together with the compiled EVM code is sent to the gas analyzer. A technical description of all phases

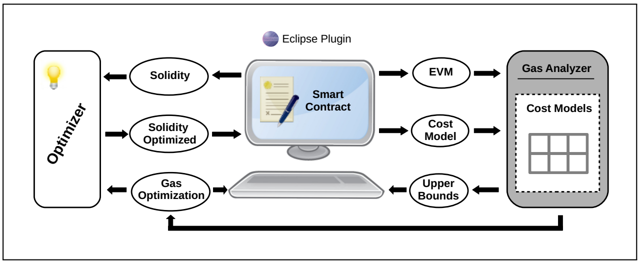

**Fig. 1.** Overview of GASOL's components that comprise a gas analysis for EVM smart contracts is given in [8]. Basically, the analyzer uses various tools [3,7] to extract the CFGs and decompile them into a high-level representation from which upper bounds (UB) are produced by using extensions of resource analyzers and solvers [4,5]. However, in our basic gas analyzer named GASTAP [8], there was only one cost model to compute the overall gas consumption of the function (including the opcode and memory gas costs [27]), while GASOL is an extension of GASTAP that introduces optimization, a wide variety of analysis options to define novel cost models, and an Eclipse plugin. The UBs are provided to the user in the console as well as in markers for functions within the Eclipse editor. If the user had selected the optimization option, the analyzer detects potential sources of optimization and feeds them to the optimizer to generate an optimized Solidity function within a new file.

Fig. 2 displays our Eclipse plugin that contains a fragment of the public smart contract ExtraBalToken [1] used as running example. We can see its six state variables and its function fill that we will analyze and optimize. The right side window shows GASOL's configuration options to set up the *cost model*:
*(i) Type of resource (gas/instructions)*: by selecting *gas*, we estimate the gas consumption according to the gas model in [27] (hence, use GASOL as a gas analyzer); by selecting *instructions*, we estimate the number of bytecode instructions executed (using GASOL as a standard complexity analyzer).
*(ii) Type of instructions*: allows selecting which instructions (or group of instructions) will be measured as follows.
- *All*: every bytecode instruction will be measured. For instance, by selecting gas in (i), the function fill, and this option, we obtain as gas bound: $1077 + 40896 \cdot data$. Besides, by using this option, GASOL also yields the so-called memory gas (see[27]): $3 \cdot (data+5) + \left\lfloor \frac{(data+5)^2}{512} \right\rfloor$. The analyzer abstracts arrays by their length, hence, these bounds are functions of the *length of the input array* (denoted as *data*) and can be used, e.g., to determine precisely how much gas is necessary to run a transaction that executes this function.
- *Gas-family*: [27] classifies bytecode instructions according to their gas consumed in six groups: zero, base, verylow, low, mid and high. Instructions that do not belong to any of the previous groups are considered as single families. This option provides the cost due to each gas-family separately and, by using the filter in (iii), we can type the name of the desired group(s). As an example, for the function fill using gas in (i), we obtain gas bounds $297 + 315 \cdot data$ and $16 + 8 \cdot data$ for the gas-families verylow and mid, resp.

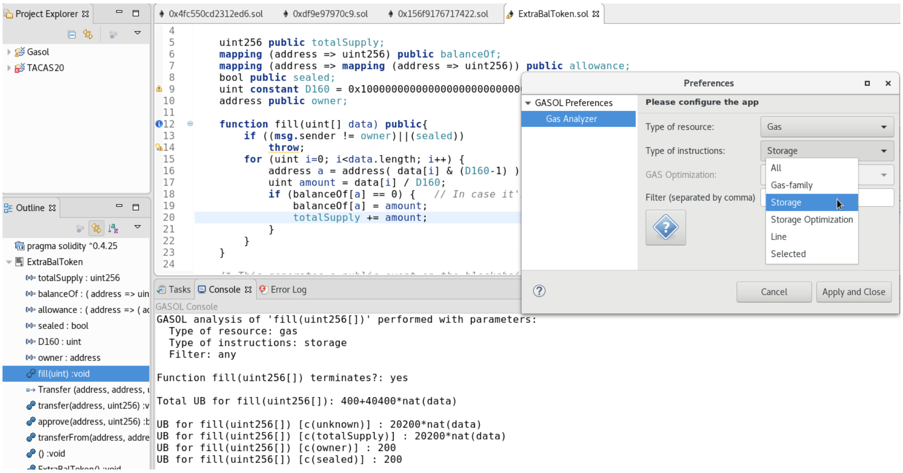

**Fig. 2.** Excerpt of smart contract `ExtraBalToken` in Solidity within Eclipse plugin.

- *Storage*: only the instructions that access the storage (namely bytecodes `SLOAD` and `SSTORE`) are accounted. The gas bounds displayed within the Eclipse console in Fig. 2 correspond to this setting, where we can see that the gas due to the access of each basic storage variable is shown separately. The first row `unknown` accumulates the gas of all accesses to non-basic types (data structures) as we still cannot identify them. By comparing this storage gas with the overall gas bound shown above for *All*, we can observe that most of the gas consumed by the function is indeed dominated by the storage (namely 40.000 out of 40.896 at each loop iteration) and it is thus a target for optimization (see Sec. 3).

- *Storage-optimization*: it bounds the number of `SLOAD` and `SSTORE` instructions executed by the current function (excluding those in transitive calls). It is the cost model that is used to detect and carry out the optimization described in Sec. 3. Thus, it is the only selection that enables the *Gas optimization* that appears as third option, and forces the selection of "instructions" as type of resource in (i). We obtain for the state variable `totalSuply` the bound: $2{\cdot}data$, which captures that we execute two accesses (one read, one write) to field `totalSuply` at each loop iteration.

- *Line*: this option allows specifying the line number (of the `Solidity` program) whose cost will be measured, and the remaining lines will be filtered out. For instance, if the line number specified in the filter (iii) is 17, i.e., the Solidity instruction: uint amount = data[i]/D160, the obtained gas bound is $3{+}97{\cdot}data$. In the absence of number in the filter, the bounds are given separately for all program lines. This option is intended to help the programmer in improving the gas consumption of her code by trying out different implementation options and comparing the results.

- *Selected*: allows computing the consumption associated to each different EVM instruction separately. For instance, if we select the bytecode instructions `MLOAD` and `SHA3`, we obtain the gas bounds $6{+}15{\cdot}data$ and $84{\cdot}data$ resp. As in the previous option, the filter allows the user to select the instructions of interest and filter out the remaining.

*(iii) Filter*: this is a text field used to filter out information from the UBs. For *gas-family*, the user can specify low, mid, etc. For *storage*, it allows specifying the name of the basic field(s) whose storage will be measured. For *line* and *selected*, we can type the line numbers and names of bytecode instructions of interest. Once all options have been selected, we have set up a cost model that is sent together with the EVM code to the gas analyzer and, after analysis, it outputs an UB for the selected function w.r.t. the cost model activated by the options. This UB is displayed, as shown in Fig. 2 in the console of the Eclipse plugin, and also within markers next to the function definition.

## 3 Gas Optimization using Gasol

The information yield by the gas analysis is used in Gasol to detect potential optimizations. Currently, the optimization target is the reduction of the gas consumption associated to the usage of storage. In particular, we aim at replacing multiple accesses to the same (global) storage data within a fragment of code (each write access costs 20.000 in the worst case and 5.000 in the best case) by one access that copies the data in storage to a (local) memory position followed by accesses to such memory position (an access to the local memory costs only 3) and a final update to the storage if needed. The cost model number of instructions for storage-optimization described in Sec. 2 allows us to detect such storage optimizations, namely for each different field, if we get a bound that is different from one, we know that there may be multiple accesses to the same position in the storage and we try to replace them by gas-efficient memory accesses. Our transformation is done at the level of the Solidity code, by defining a local variable with the same name as the state variable to transform, and introducing setter and getter functions to access the storage variable. Currently, we can transform accesses to variables of basic types, in the future, we plan to extend it to data structures (maps and arrays). The number of instructions bound for field `totalSupply` is $2 \cdot data$ (hence $\neq 1$), and our optimization of `fill` is:

```
function fill (uint [] data) {
  uint256 totalSupply = get_field_totalSupply (); if ((msg.sender != owner)||(sealed))
     throw;
  for (uint i=0; i<data.length; i++) {
    address a = address( data[i] & (D160-1) );
    uint amount = data[i] / D160;
    if (balanceOf[a] == 0) {
      balanceOf[a] = amount;
      totalSupply += amount;
    }
  }
  set_field_totalSupply (totalSupply);
}
```

The gas bound (using the option *All*) for the optimized fill yield by Gasol is $21368 + 20674 \cdot data$, which means that, assuming the worst case for write access to storage, the gas consumed inside the loop is 49.45% smaller than the one for the original fill function (the memory gas does not change). Note that, even if we consider the best case of 5.000 for write access to storage for the accesses we have optimized, the gas reduction is still around 20%. This is, in fact, what we have manually estimated using the actual data of the 82 times this function has been executed in the Ethereum blockchain, achieving with Gasol a total saving of almost 60M gas. As our transformation is local to the function, in order to be sound, we check that the transformed global data is not being accessed by transitive calls. For instance, if there was a call to another function from function fill that accesses `totalSupply`, we would not transform it. Besides, for efficiency, we check if all accesses are read (bytecode SLOAD) and, in such case, we do not need to invoke the setter at the end (and avoid an unnecessary write access).

## 4 Related Tools and Conclusions

Numerous tools are being developed to catch different types of vulnerabilities of smart contracts [20,16,22,19,17,26,18,10,15,9]. As mentioned in Sec. 1, the Solidity compiler `solc` is not able to give any gas estimation for the running example, as its gas consumption is not constant. Therefore, new gas analysis tools are being developed to detect potential gas related vulnerabilities and to infer bounds in these complex situations. The purpose of the Gasper and MadMax tools is precisely the detection of gas related vulnerabilities. MadMax [14] focuses on identifying control- and data-flow patterns inherent for the gas-related vulnerabilities, thus, it works as a bug-finder, rather than as a gas analyzer like Gasol. Similarly, Gasper identifies gas-costly programming patterns [12] by matching specific control-flow patterns and using SMT solvers and symbolic computation. Thus, it is an optimization detector, not an automatic optimizer as Gasol. The recently developed `ebso` tool [24] also aims at optimizing the gas consumption of EVM code. In contrast to Gasol, `ebso`'s optimizations are limited to a basic block level, while our transformation might involve several blocks of the CFG and would not be achievable by `ebso`'s approach. Also, `ebso` is not guided by the results of an automatic resource analysis which can capture the expensive storage patterns as in our case. Instead it is based on a full exploration of all possible alternative instructions (within the considered block) that would lead to the same result and consume less gas. They have obtained a number of rewrite rules that define sequences of bytecode instructions that can be replaced by equivalent ones that consume less. We could easily incorporate such basic block replacement optimizations within our tool, and it is part of our agenda.

The approach of [21], like ours, aims at inferring precise gas bounds. Their approach is based on symbolically enumerating all execution paths [11] and unwinding loops to a limit. Instead, using resource analysis, Gasol infers the maximal number of iterations for loops and generates accurate gas bounds which are valid for any possible execution of the function and not only for the unwound paths. The approach by Marescotti *et al.* has not been implemented in the context of EVM and a tool like Gasol has not been delivered. An orthogonal line of work with ours is the construction of resource-oriented attacks [23] that exploit the weaknesses of the EVM gas model. Gasol's cost models could help detect this resource-oriented attacks by estimating the number of executed bytecode instructions (very high) and their associated gas consumption (very low).

Finally, there is a tendency to define new languages (see Scilla [25], Michelson [2]) for programming smart contracts that provide certain safety guarantees, e.g., Scilla [25] provides predictable gas consumption by disallowing general recursion and while-loops. However, Ethereum is today the most widely used blockchain, and Solidity the most popular programming language to write Ethereum smart contracts, for which a gas analyzer+optimizer is of clear relevance.
